# Quantitative Framework for Model Evaluation in Microbiology Research Using *Pseudomonas aeruginosa* and Cystic Fibrosis Infection as a Test Case

**DOI:** 10.1128/mBio.03042-19

**Published:** 2020-01-14

**Authors:** Daniel M. Cornforth, Frances L. Diggle, Jeffrey A. Melvin, Jennifer M. Bomberger, Marvin Whiteley

**Affiliations:** a School of Biological Sciences, Georgia Institute of Technology, Center for Microbial Dynamics and Infection, Georgia Institute of Technology, Atlanta, Georgia, USA; b Department of Microbiology and Molecular Genetics, University of Pittsburgh School of Medicine, Pittsburgh, Pennsylvania, USA; c Emory–Children’s Cystic Fibrosis Center, Atlanta, Georgia, USA; Johns Hopkins Bloomberg School of Public Health

**Keywords:** *Pseudomonas aeruginosa*, model, transcriptomics, cystic fibrosis, infection

## Abstract

Laboratory models are a cornerstone of modern microbiology, but the accuracy of these models has not been systematically evaluated. As a result, researchers often choose models based on intuition or incomplete data. We propose a general quantitative framework to assess model accuracy from RNA sequencing data and use this framework to evaluate models of Pseudomonas aeruginosa cystic fibrosis (CF) lung infection. We found that an *in vitro* synthetic CF sputum medium model and a CF airway epithelial cell model had the highest genome-wide accuracy but underperformed on distinct functional categories, including porins and polyamine biosynthesis for the synthetic sputum medium and protein synthesis for the epithelial cell model. We identified 211 “elusive” genes that were not mimicked in a reference strain grown in any laboratory model but found that many were captured by using a clinical isolate. These methods provide researchers with an evidence-based foundation to select and improve laboratory models.

## Abstract (precis)

Laboratory models have become a cornerstone of modern microbiology. However, the accuracy of even the most commonly used models has never been evaluated. Here, we propose a quantitative framework based on gene expression data to evaluate model performance and apply it to models of Pseudomonas aeruginosa cystic fibrosis lung infection. We discovered that these models captured different aspects of P. aeruginosa infection physiology, and we identify which functional categories are and are not captured by each model. These methods will provide researchers with a solid basis to choose among laboratory models depending on the scientific question of interest and will help improve existing experimental models.

## INTRODUCTION

For over a century, microbiologists have relied on laboratory models to study pathogenic bacteria (1, 2). Due to obvious ethical prohibitions on human experimentation, laboratory infection models have become a cornerstone in bacterial pathogen research. These models range in complexity from standard laboratory media, to *in vitro* models specifically designed to mimic infection, to the most complex class of models, animal hosts.

There are typically a range of laboratory models used to study any given infection type. These model systems are selected based on a balance between each model’s perceived strengths and limitations. For instance, *in vitro* models are often inexpensive and highly controllable, whereas animal models are thought to capture important aspects of human pathogenesis, such as host immunity and tissue structure, which can be difficult to reproduce *in vitro* (2). The accuracy of a laboratory model is likely shaped by several factors, including the bacterial genotype used (3), the chemical and physical environment the bacteria are exposed to (4), and even the mode of inoculation (5). Although most researchers are aware of these considerations, there is no clear framework for deciding which model best addresses a given research question. Until recently there have been insufficient data on bacterial behavior and physiology in clinical infections to effectively evaluate laboratory model performance, and beyond this limitation, there has been no formalized framework to do so. The lack of a systematic framework for model selection has left researchers to rely on intuition or *ad hoc* rationalizations for selecting their model.

We address this issue by proposing a framework to evaluate the accuracy of human infection models using RNA sequencing (RNA-seq) data. RNA-seq provides a snapshot of pathogen gene expression, giving a rare glimpse of bacterial behavior and physiology in a natural, unmanipulated human infection environment. The present study was motivated by a desire to evaluate the accuracy of models used to study Pseudomonas aeruginosa infection of the lungs of cystic fibrosis (CF) patients. CF is a recessive genetic disease caused by mutations in the gene encoding the cystic fibrosis transmembrane conductance regulator (CFTR), an ion channel that conducts chloride and bicarbonate across epithelial cell membranes. Mutations in CFTR result in accumulation of viscous mucus (sputum) in an individual’s lungs, which is subsequently colonized by P. aeruginosa and other bacteria (6, 7). After colonization, P. aeruginosa can use lung sputum as a carbon and energy source (8–10), allowing it to grow to high densities and persist throughout a patient’s life.

One of the challenges to studying CF infection biology has been the lack of robust *in vivo* and *in vitro* model systems. Numerous murine models have been developed to study P. aeruginosa lung infection, many of which use mice with wild-type CFTR (11–16). Models using mice with mutant CFTR have also been developed (17), although these models generally show high-level resistance to P. aeruginosa infection, which is likely due to specific aspects of mouse lung physiology (18). Several *in vitro* models have also been developed, including one in which P. aeruginosa is inoculated on the apical surface of CFTR mutant human airway epithelial cells that have been differentiated at the air-liquid interface (19). While clearly a simplified model compared to the mouse, this model has been used to study coinfection (20) and has the advantage of studying the host-pathogen interaction while maintaining experimental versatility. Other *in vitro* model systems do not include host cells but are instead meant to mimic the chemical and physical aspects of expectorated CF sputum. These models include a defined synthetic CF sputum medium (SCFM2) that both mimics the chemistry and viscosity of CF sputum (9, 10, 21, 22). P. aeruginosa grown in synthetic sputum has a similar gene expression signature to P. aeruginosa grown in human CF sputum *in vitro* (22), and P. aeruginosa requires similar genes to grow in SCFM2 as it does in expectorated human CF sputum (23).

Though the relevance of the models outlined above has been rationalized for the study of CF infection, it is likely that these models do not capture all aspects of P. aeruginosa physiology in the CF lung. To further explore this possibility, we analyzed P. aeruginosa transcriptomes from human CF sputum samples taken immediately following expectoration from two CF clinics, one in Copenhagen, Denmark, and one in Atlanta, GA. We propose a computational framework that uses these transcriptomic data, along with transcriptomic data from CF infection models, to assess the overall model accuracy for reproducing P. aeruginosa CF lung physiology. In addition to assessing overall accuracy, we used gene expression to infer P. aeruginosa biological functions that are and are not reproduced in each model. Our results revealed that SCFM2 and an *in vitro* CF epithelial cell model mimicked the P. aeruginosa transcriptome in expectorated human sputum better than other models tested, including a mouse lung infection model currently used to study CF lung infections. Although the models differed in overall accuracy, we found that each model reproduces particular P. aeruginosa functions present in CF lung infections and, further, that some functions were not reproduced by any models we tested. The framework we propose is a step toward a grounded, evidence-based approach for selection of an infection model based on the function(s) of interest and for identifying strategies for model improvement.

## RESULTS

### Acquisition and mapping of *P. aeruginosa* RNA-seq reads from expectorated human CF sputum.

We analyzed P. aeruginosa transcriptomes from 20 CF sputum samples collected from 19 clinically stable patients. Samples were stored in RNAlater (Invitrogen) immediately after expectoration until RNA extraction. Seven of these samples were collected from a Danish clinic and analyzed previously (24), and 13 samples were collected from the Center for Cystic Fibrosis and Airway Disease Research in Atlanta, GA, and are being analyzed for the first time here. Because CF lung infections are often polymicrobial, after trimming adapters, we mapped all RNA-seq reads to 54 bacterial species that we previously identified as present among CF sputum samples using CLARK (24) (see Data Set S1 in the supplemental material). We initially mapped against one or more genomes from each of these non-P. aeruginosa species (totaling 101 genomes). We ignored reads that mapped to these non-P. aeruginosa species in order to avoid attributing differences in transcript frequencies to P. aeruginosa when another bacterium was potentially responsible. This approach was conservative because P. aeruginosa-mapping reads that closely resembled sequences of other species were discarded, but it gives us confidence that the differences we observed were caused by differences in P. aeruginosa RNA levels. We mapped all remaining reads to genes of the well-annotated P. aeruginosa reference strain PAO1. All samples used in our analyses had reads mapping to at least 4,000 of PAO1’s 5,570 genes (Data Set S1). We similarly analyzed 67 published transcriptomes of P. aeruginosa grown in a range of laboratory models, including lysogeny broth (LB), MOPS (morpholinepropanesulfonic acid)-succinate (4), SCFM2 (23), a mouse lung infection model (13), and an *in vitro* CFTR ΔF508 CFBE41o^–^ mutant polarized epithelial cell model (19, 20) (see Data Set S1 for a description of all analyzed samples).

10.1128/mBio.03042-19.7 DATA SET S1 Sample information and decoy species list. SRA accessions are given for all published samples, the transcriptomes analyzed first in this paper are available under acession number PRJNA576508. (Sheet 1) Clinical and sequencing information on the human samples used in this study. The blue highlighted text indicates that these two samples came from the same patient. (Sheet 2) *In vitro* and mouse samples we analyzed in this study. (Sheet 3) Non-P. aeruginosa “decoy” species genomes to which we initially mapped our sequencing reads. Download Data Set S1, XLSX file, 0.1 MB. Copyright © 2020 Cornforth et al. 2020 Cornforth et al. This content is distributed under the terms of the Creative Commons Attribution 4.0 International license.

### *P. aeruginosa* transcriptome from expectorated human CF sputum is distinct from that from laboratory models.

**FIG 1:**
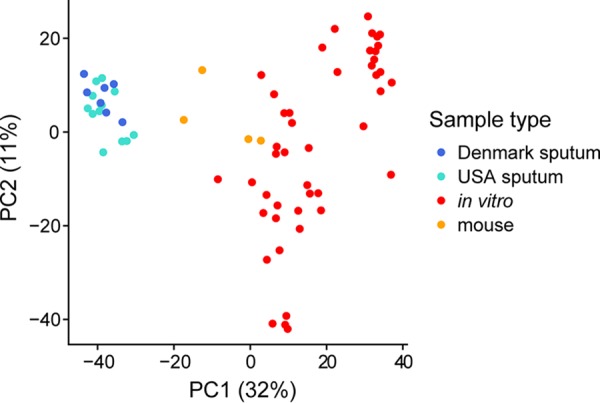
P. aeruginosa transcriptomes from human CF sputum cluster distinctly from *in vitro* and mouse acute lung transcriptomes using principal-component analysis. The analysis is based on 2,606 genes that had at least one read mapping to them in all samples (see Data Set S2 for this shared gene list). For clarity, in all laboratory conditions, only one replicate from each experiment is shown, which is identified in Data Set S1 in the supplemental material.

To assess overall relationships among the P. aeruginosa transcriptomes from expectorated CF sputum and model systems, we first performed principal-component analysis (PCA) (Fig. 1). We restricted this initial analysis to 2,606 genes for which all transcriptomes had at least one read mapping to avoid biasing results due to the presence or absence of certain genes across strains. The PCA results in Fig. 1 show a clear separation between human P. aeruginosa CF sputum transcriptomes and those of laboratory models (see Fig. S1 for a scree plot showing the total variance in the data explained by each principal component). The separation between human and *in vitro* transcriptomes occurs primarily along the first principal component, which accounts for approximately 32% of the overall variance in the transcriptomes. Genes contributing most to the differences in the first principal component included several genes with higher expression in the CF sputum transcriptomes than in *in vitro* ones, such as *llda* (l-lactate dehydrogenase), genes involved in alginate production (*algA* and *alg8*), and a heme uptake receptor (*phuR*). In addition, genes with higher expression *in vitro* also contributed significantly to the first principal component included an aerotaxis methyl-accepting chemotaxis protein (*aer2*), elastase (*lasB*), and a gene encoding a quinolone signal response protein (*pqsE*).

10.1128/mBio.03042-19.1 FIG S1 Scree plot of the sample transcriptomes used to make the PCA in Fig. 1. The scree plot displays how much variance is explained by each principal component based on the 2,606 genes for which all samples had at least one read mapping to them (see Data Set S2 for this shared gene list). As in the PCA in Fig. 1, for laboratory conditions, only one replicate from each experiment was used to calculate the variances (identified in Data Set S1). Download FIG S1, PDF file, 0.05 MB. Copyright © 2020 Cornforth et al. 2020 Cornforth et al. This content is distributed under the terms of the Creative Commons Attribution 4.0 International license.

**FIG 2:**
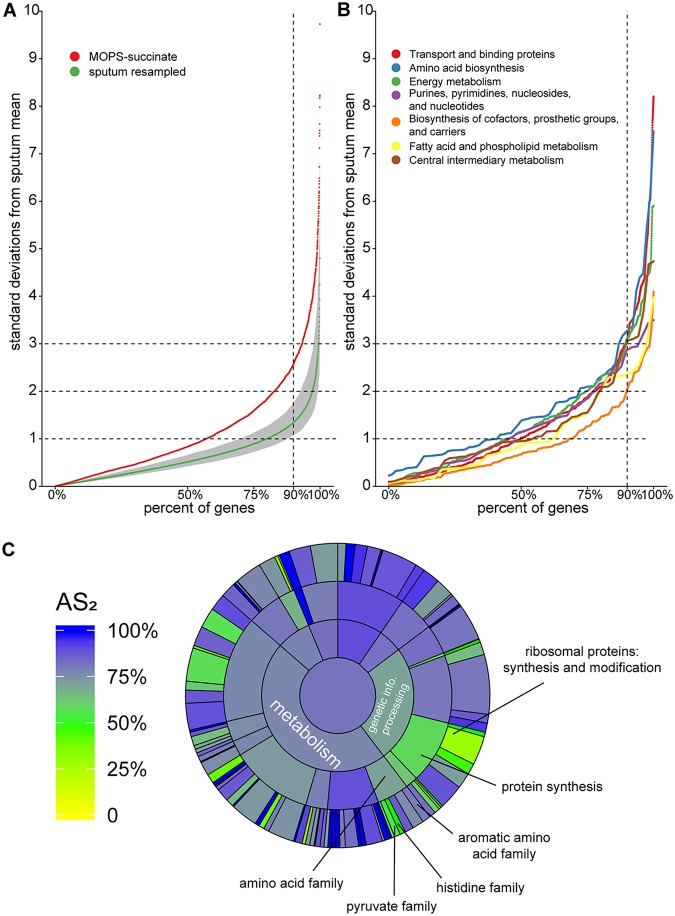
Genome-wide accuracy metric for strain PAO1 grown planktonically in MOPS-succinate. (A) Percentages of PAO1 genes (*x* axis) within different numbers of standard deviations of the mean expression in human CF sputum (*y* axis). For each gene, the mean and standard deviation of normalized read counts among the sputum samples were calculated. The mean expression for each gene in MOPS-succinate was then determined, and a z-score (the number of standard deviations each gene is from the mean expression in CF sputum) was calculated. For reference, we performed the same procedure on 100 randomly resampled pairs of human CF sputum samples to provide a robust assessment of the variance in these samples (sputum resampled). The mean and 95% confidence interval for each of these resampled values for each gene are shown. Any genes with values over 10 standard deviations from the sputum mean are not shown. (B) An accuracy metric was calculated for PAO1 grown in MOPS-succinate for all TIGRFAM *“*metabolism*”* meta roles. (C) AS_2_ for each TIGRFAM meta role, main role, and sub role category for PAO1 grown in MOPS-succinate. The color in the middle represents the AS_2_ for all PAO1 genes (those with or without TIGRFAM designations). The next level out from the middle of the circle contains “meta roles,” the next contains “main roles,” and the outermost layer contains “sub roles.” The area of each category is proportional to the number of genes in that category.

**FIG 3:**
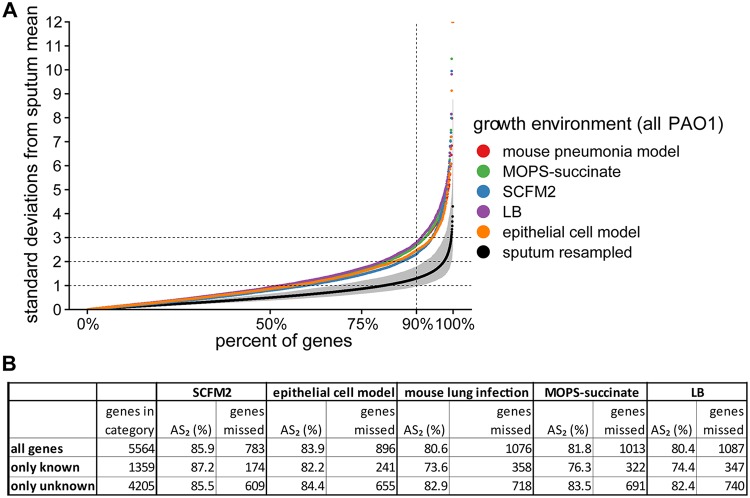
Genome-wide accuracy metric for PAO1 grown in five model systems. The model systems include an acute mouse pneumonia model, planktonic growth in MOPS-succinate, SCFM2 with no shaking, planktonic growth in LB, and growth in a CF airway epithelial cell model. (A) Percentage of PAO1 genes (*x* axis) within different numbers of standard deviations of the mean expression in human CF sputum (*y* axis) for each model, calculated as described in Fig. 2A. For reference, we performed the same procedure on 100 randomly resampled pairs of human CF sputum samples to provide a robust assessment of the variance in these samples (sputum resampled). The mean and 95% confidence interval of these resampled values for each gene is shown. Any genes with values over 12 standard deviations from the sputum mean are not shown. (B) Table containing the accuracy scores (AS_2_) and number P. aeruginosa genes in each model not within two standard deviations of the mean in CF sputum (genes missed). The genes are divided into “all genes,” “known,*”* and “unknown.” “Unknown” refers to genes that have a TIGRFAM “main role” with either no category designation, have a “main role” annotated as “unknown function,” or are not annotated in the TIGRFAM database. We calculated pairwise *t* tests between sample types using genome-wide AS_2_ scores for individual replicates in each sample type, with a Bonferroni adjustment for multiple tests. The most significant comparisons between model types were for SCFM2 compared to LB (*P* = 0.015), the mouse pneumonia model (*P* = 0.018), and MOPS-succinate (*P* = 0.073). All other model pairs had adjusted *P* values of >0.2.

10.1128/mBio.03042-19.8 DATA SET S2 Z-score accuracy assessment for all genes across all tested models, list of genes used in Fig. 1, and differential expression analysis between Atlanta and Copenhagen CF clinics. (Sheet 1) z-Scores for all the models tested for each PAO1 orthologous gene. Scores were calculated using the mean normalized expression among model replicates as in Fig. 2 and 3. (Sheet 2) List of 2,606 genes for which all sample transcriptomes had at least one read (used in the PCA in Fig. 1). (Sheet 3) Differential expression analysis between sputum transcriptomes originating from the Atlanta versus Copenhagen clinics, performed using DESeq2 with default parameters. (Sheet 4) List of the 211 elusive genes that were not captured by any model. Download Data Set S2, XLSX file, 0.7 MB. Copyright © 2020 Cornforth et al. 2020 Cornforth et al. This content is distributed under the terms of the Creative Commons Attribution 4.0 International license.

While sputum samples from the United States and Denmark clinics were shown to be similar in Fig. 1, a PCA conducted only among the sputum samples demonstrates a separation between transcriptomes from the two clinics (see Fig. S2 in the supplemental material). Consistent with this separation, differential expression analysis indicates several differences in P. aeruginosa gene expression between the two clinic populations (see Data Set S2 in the supplemental material). The genes with the largest expression differences between clinics had no reads mapping for many samples, such as the AraC-like transcriptional regulator *vqsM* that had no reads mapping to it in any sputum transcriptomes except for three samples from the U.S. clinic. Because it is difficult to determine whether these differences were caused by strain differences in gene content rather than differences in regulation, we restricted the differential expression analysis to the 3,225 genes that had at least one read mapping to them in all CF sputum transcriptomes. The most differentially expressed genes with this approach were often not consistently different between the clinics. For instance, the gene most differentially expressed between the two clinics was the autoinducer synthase *lasI* (expressed at ∼26-fold-higher levels in the Denmark samples, *P* = 9.5 × 10^−5^), which encodes a protein that produces a quorum sensing signal in P. aeruginosa. However, the magnitude of this effect is primarily due to relatively high expression in just two of the seven Denmark samples. Other highly differentially expressed genes include *dctA* (C4-dicarboxylate transport protein, higher in the Denmark sputum samples), *lptG* (lipopolysaccharide export system permease protein LptG, higher in the Denmark sputum samples) and *flgI* (flagella P-ring protein precursor, higher in the U.S. sputum samples). We also found that the beta-lactamase precursor *ampC* was expressed at 6.7-fold-higher levels among the Denmark samples.

10.1128/mBio.03042-19.2 FIG S2 PCA of normalized gene counts among all 20 human CF sputum samples without additional sample types, based on the 2,606 genes analyzed in Fig. 1. There is a clear separation between the transcriptomes from the two clinics that was not present in the PCA in Fig. 1 due to the influence of laboratory model samples on the principal components. Download FIG S2, PDF file, 0.05 MB. Copyright © 2020 Cornforth et al. 2020 Cornforth et al. This content is distributed under the terms of the Creative Commons Attribution 4.0 International license.

### *P. aeruginosa* metabolism in CF sputum.

One of our major interests is microbial metabolism during infection, and the 20 CF sputum transcriptomes provided an opportunity to examine P. aeruginosa metabolism in the CF lung. To accomplish this, we compared the CF sputum transcriptomes to a well-characterized laboratory environment in which cellular metabolism is well understood. Specifically, we compared the sputum transcriptomes to transcriptomes of the reference strain PAO1 grown planktonically with vigorous shaking to mid-logarithmic phase in a well-defined MOPS-buffered medium with succinate as a sole carbon source (4). The sputum samples showed several indications of lower oxygen levels compared to growth in MOPS-succinate, including higher expression of denitrification operons *nor*, *nir*, *nar*, *nos*, and *nap*. In addition the sputum transcriptomes displayed relatively high expression of genes encoding the high-affinity cyanide insensitive terminal oxidase (*cioAB*). However, genes encoding the low-affinity cytochrome *o* ubiquinol oxidase (*cyoABCDE*), which is not critical for growth in low oxygen concentrations (>2%), were also highly upregulated in sputum. Decreased expression of genes encoding enzymes for several decarboxylation steps of the tricarboxylic acid cycle (via *sucA*, *sucC*, and *lpd*), together with increases in *aceA* and *glcB* expression, suggest an increased flux in carbon through the glyoxylate shunt among sputum samples. Also, consistent with previous work, we observed higher expression of *lldA* in sputum samples, which is involved in l-lactate catabolism, as well as greater expression of zinc uptake and transport genes (*znuB*, *znuC*, and *zur*) (24).

To identify additional differences in metabolic activity between the MOPS-succinate and sputum transcriptomes, we performed gene set enrichment analysis (GSEA) with Pseudocyc gene annotations (25) (see Materials and Methods for details). Compared to those of the MOPS-succinate samples, the sputum sample transcriptomes showed enrichment of genes with lower expression in sputum among pathways involved in the synthesis of several amino acids and intermediate products including threonine (*P* = 3.5 × 10^−3^), homoserine (*P* = 2.9 × 10^−3^), leucine (*P* = 1.7 × 10^−2^), isoleucine (*P* = 1.9 × 10^−2^), histidine (*P* = 7.0 × 10^−3^), and glutamate (*P* = 2.9 × 10^−3^). We also performed an enrichment analysis using TIGRFAM “function” categories and discovered that genes encoding TonB dependent receptors were enriched for greater expression in the sputum, including siderophore receptors (*P* = 1.2 × 10^−3^) (consistent with an increased expression of genes involved in synthesis and regulation of the siderophores pyoverdine and pyochelin), as well as heme/hemoglobin/transferrin/lactoferrin receptors (*P* = 4.2 × 10^−3^).

In addition to differences in metabolic activity between the sputum samples and MOPS-succinate samples, the DNA-damage stress response regulator *lexA* and genes encoding reactive oxygen species-scavenging enzymes (*ahpB*, *ahpC*, *ahpF*, *katB*, *sodM*, and *ohr*) were expressed at substantially higher levels in the sputum samples. Consistent with previous work (3), genes for choline and l-carnitine degradation to the osmoprotectant glycine betaine (*opuCD*, *betA*, *betB*, and *cdhC*) were highly expressed in sputum, as well as the transcriptional repressor *betI*, but *gbt*, which is required to use choline and glycine betaine as a carbon source, was expressed less in sputum than in MOPS-succinate. This result may indicate that glycine betaine is primarily used as an osmoprotectant rather than for carbon and nitrogen acquisition (3).

### Framework to evaluate infection models.

While differential expression analysis was useful in understanding basic differences in gene expression between CF sputum and specific laboratory conditions such as MOPS-succinate, our goal was to develop an explicit framework to evaluate how well different aspects of any experimental model system mimic the “target” system (P. aeruginosa in human CF sputum in this case). An ideal evaluation framework should have the following characteristics: (i) it should provide a simple biological interpretation that allows for a straightforward comparison of models, (ii) it should be able to determine both the genome-wide accuracy of the experimental model and the model’s accuracy for any functional category of interest, and (iii) it should not be inherently dependent on the number of available samples (as with differential expression analysis, where additional samples lead to increased statistical power and thus more genes being called as differentially expressed).

We propose a framework based on the number of standard deviations in normalized expression for each gene the model is from the mean expression among target transcriptomes. In the first step, we calculate the mean and standard deviation of normalized read counts for each gene among target transcriptomes. Then, for each model system, we average the expression levels of each gene among the replicates and calculate a z-score, which is the number of standard deviations in expression that the model is above the mean observed among the target transcriptomes. We use the absolute value of each gene’s z-score as an indication of how similarly the gene is expressed between the model and target. From this perspective, one can ask for any model system, what fraction of a study organism’s genes are within two standard deviations (for example) of the mean expression in the target transcriptomes or, similarly, one can ask how many standard deviations from the target transcriptome mean are required in order to include 95% (for example) of the organism’s genes. These perspectives are complementary; however, in practice we find the former to be more intuitive and here define an “accuracy score” based on it. A model’s accuracy score (AS) is the fraction of the organism’s genes that are within a specified number of standard deviations from the target. For example, if a model has an AS_2_ of 80%, then the expression of 80% of the model’s genes fall within two standard deviations of the means of the gene among the target transcriptomes. An AS_2_ of 90% would indicate a more accurate model because 90% of the model’s genes are expressed at levels within two standard deviations of the target’s means.

We first used this framework to provide a genome-wide evaluation of the accuracy of P. aeruginosa strain PAO1 grown planktonically in MOPS-succinate as a model for CF lung infection. Again, we began with MOPS-succinate since it provides a well-defined growth condition for our initial comparisons. Figure 2A shows the percentage of genes whose average normalized expression among replicates is within each standard deviation cutoff of interest. The AS_2_ of PAO1 in MOPS-succinate is approximately 82% (meaning 82% of the PAO1 genome has expression in MOPS-succinate that is within two standard deviations of the expression in the sputum samples). As a control, we also calculated the performance of resampled human CF sputum P. aeruginosa transcriptomes by randomly choosing pairs of the 20 CF sputum transcriptomes, averaging the normalized expression for each gene and then calculating the standard deviations from the mean expression in these genes calculated using all clinical sputum samples except the chosen two being treated as the “model.” We repeated this process 100 times to obtain a mean value and a 95% confidence interval (Fig. 2A). The average AS_2_ for these resampled human CF sputum transcriptomes was 97%, indicating that randomly chosen human CF sputum transcriptomes performed significantly better than the MOPS-succinate transcriptomes (*P* = 1.6 × 10^−5^ [*t* test]).

This approach can be applied to any functional category, pathway, or individual gene of interest. To obtain functional resolution of the accuracy assessment, we used the TIGRFAM functional gene annotation database, wherein annotated genes are assigned to have at least one “main role,” “sub role,” and “function,” forming a hierarchy of increasing specificity. We first focused on the accuracy of metabolic genes in PAO1 growing in MOPS-succinate (Fig. 2B). This analysis demonstrates that MOPS-succinate mimics P. aeruginosa gene expression in sputum for some metabolic functions such as “biosynthesis of cofactors, prosthetic groups, and carriers,” with an AS_2_ of approximately 89%, whereas others such as amino acid biosynthesis (AS_2_ = 71%) are poorly mimicked. The latter aligns well with our differential expression and enrichment analysis above, which indicated that several amino acid biosynthetic pathways were differentially regulated between CF sputum and MOPS-succinate.

We then took a more expansive view, calculating the model performance for the genes within every TIGRFAM category (Fig. 2C). We added an additional, overarching level to the standard TIGRFAM hierarchy called “meta roles,” as previously described (26). By calculating these values for different levels of the TIGRFAM hierarchy, we could determine whether categories were influenced by a minority of subcategories or whether genes across these categories scored similarly. For instance, we identified amino acid biosynthesis as a poor performing category (AS_2_ = 71%), and Fig. 2C shows that the sub roles “pyruvate family” (AS_2_ = 55%) and “histidine family” (AS_2_ = 50%) reduce the overall “amino acid biosynthesis” score substantially, whereas the “aromatic amino acid family” performs better (AS_2_ = 80%). Similarly, though the “protein synthesis” category scores poorly overall (AS_2_ = 58%), this poor performance is predominantly restricted to genes involved in the synthesis and modification of ribosomal proteins.

### Accuracy of several models used to study *P. aeruginosa* lung infection.

We then applied this framework to several additional experimental models: lysogeny broth (LB), a mouse pneumonia model (13), synthetic sputum medium (SCFM2) (4), and the *in vitro* CFTR ΔF508 CFBE41o^–^ mutant polarized airway epithelial cell model (27). In order to reduce the impact of strain differences on the results and assess how well a common laboratory strain captures P. aeruginosa physiology in the CF lung, we only compared data from laboratory systems inoculated with the reference strain PAO1. It has previously been shown that PAO1 strains can differ both genotypically and phenotypically (28, 29); although the MOPS-succinate (4), SCFM2, airway epithelial cell model, and LB (30) experiments used PAO1-UW (31, 32), the mouse experiments used a PAO1 strain from the Vasil lab (13). Replicates for all models clustered closely according to the z-score across the P. aeruginosa genome, indicating that these models have high reproducibility (Fig. S3).

10.1128/mBio.03042-19.3 FIG S3 PCA of genome-wide z-scores (based on mean and standard deviation of sputum samples) for each laboratory condition. The close clustering between transcriptomes of replicates from each model indicates a high similarity in genome-wide accuracy between replicates. Download FIG S3, PDF file, 0.1 MB. Copyright © 2020 Cornforth et al. 2020 Cornforth et al. This content is distributed under the terms of the Creative Commons Attribution 4.0 International license.

We began by comparing the genome-wide accuracy for all five experimental models (Fig. 3). The two models that were explicitly designed to mimic CF lung infection, SCFM2 and the CF airway epithelial cell model, had the highest raw AS_2_ values (86 and 84%, respectively) (Fig. 3B). These models were followed by MOPS-succinate (82%), then the mouse pneumonia model (81%), and finally LB (80%) (Fig. 3B). However, only SCFM2 showed statistically significant improvement over other models: LB (*P* = 0.015), the mouse pneumonia model (*P* = 0.018), and MOPS-succinate (*P* = 0.073) (pairwise *t* test using a Bonferroni adjustment for multiple tests). All models performed worse than the resampled human CF sputum (Fig. 3A), indicating that there is still room for improvement for every model. The z-scores for all genes across all models tested is available in Data Set S2.

**FIG 4:**
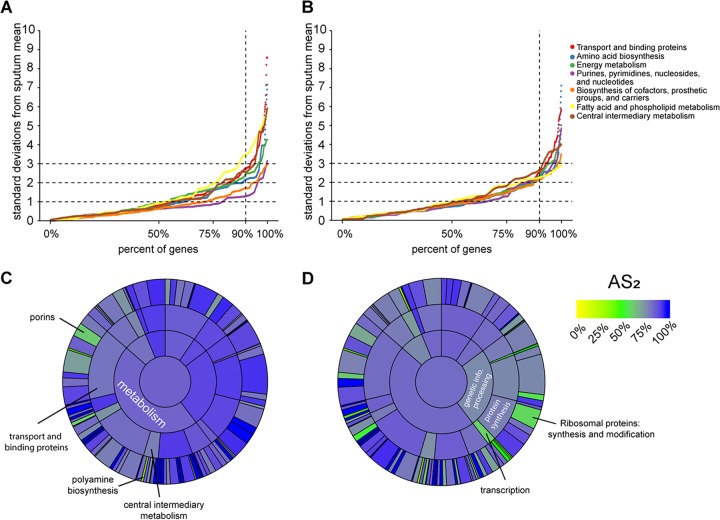
Accuracy metric for TIGRFAM subcategories for P. aeruginosa PAO1 in SCFM2 and the CF airway epithelial cell infection model. (A and B) Percentages of P. aeruginosa PAO1 genes within each TIGRFAM metabolism *“*sub role” whose mean expression in SCFM2 (A) and CF airway epithelial cell infection model (B) transcriptomes fall within different numbers of standard deviations of the mean expression in sputum samples (absolute value of z-score, calculated as described in Fig. 2A). (C and D) AS_2_ for each TIGRFAM meta role, main role, and sub role category for SCFM2 (C) and the CF airway epithelial cell infection model (D). The color in the middle represents the AS_2_ for all PAO1 genes (those with or without TIGRFAM designations). The next level out from the middle of the circle contains “meta roles,” the next contains “main roles,” and the outermost layer contains “sub roles.” The area of each category is proportional to the number of genes in that category.

Because SCFM2 was specifically designed to mimic the metabolism of P. aeruginosa growth in CF sputum, we focused on the accuracy of metabolic genes in PAO1 growing in SCFM2 (Fig. 4A). We also evaluated the other explicit CF model, the CF airway epithelial cell model, wherein the bacteria acquire nutrients partially from the airway epithelial cells (Fig. 4B). The metabolic categories that SCFM2 captured best were “purines, pyrimidines, nucleosides, and nucleotides,” which the CF airway epithelial cell model captures considerably worse. On the other hand, SCFM2 performed worst at mimicking fatty acid and phospholipid metabolism, a category for which the CF airway epithelial cell model performed markedly better. As before, we then expanded our view to all TIGRFAM categories by calculating the AS_2_ of genes within every TIGRFAM category (Fig. 4C and D). Though SCFM2 captures “transport and binding proteins” well overall (AS_2_ = 82%), some subcategories, such as “porins” are poorly mimicked (AS_2_ = 62%) (Fig. 4C). Porins were captured better in the CF airway epithelial cell model (AS_2_ = 70%), but genes involved in “synthesis and modification of ribosomal proteins” performed worse than in SCFM2 (AS_2_ of 58% versus 83%). To ensure that our analysis was not biased by sequencing depth, we repeated the basic analysis with all samples resampled down to 100,000 reads, which did not qualitatively affect our results (Fig. S4).

10.1128/mBio.03042-19.4 FIG S4 Accuracy scores calculated from randomly subsampled gene expression counts files. All transcriptomes were randomly subsampled down to 100,000 reads and then plotted as in Fig. 3 of the main text. This severe subsampling does not qualitatively impact the accuracy results across models. Download FIG S4, PDF file, 0.2 MB. Copyright © 2020 Cornforth et al. 2020 Cornforth et al. This content is distributed under the terms of the Creative Commons Attribution 4.0 International license.

### Improvements in accuracy by combining models.

**FIG 5:**
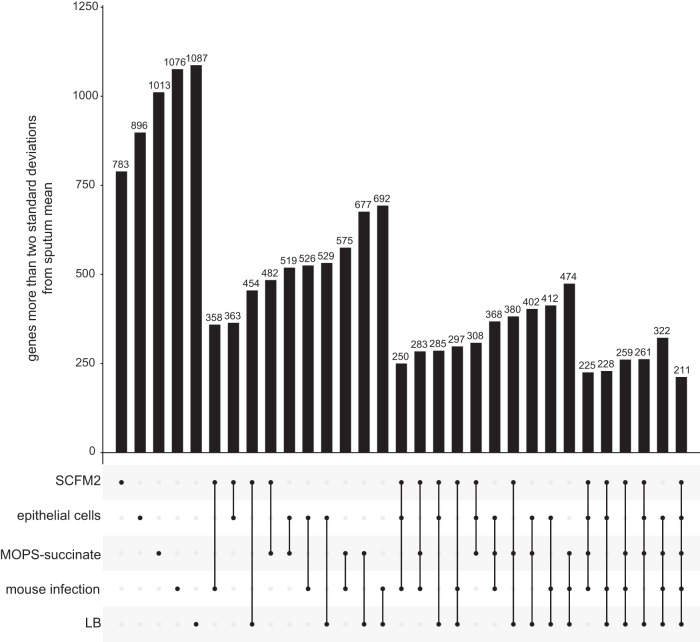
Number of genes whose expression is not within two standard deviations of the CF sputum transcriptome means for any infection model among each possible combination of models. For example, the sixth bar indicates that 358 genes have expression outside two standard deviations from the mean of sputum sample transcriptomes for both SCFM2, as well as for the mouse pneumonia model. The final column indicates that 211 genes have expression outside two standard deviations in all of the five evaluated models.

Figure 3 shows that, as expected, no tested model perfectly mimics the gene expression of P. aeruginosa CF sputum infections. An obvious question is whether each model misses the same genes, or whether each model has different limitations. This is a critical question since it will determine whether a CF infection researcher can study nearly any gene of interest by selecting the appropriate PAO1 model. To answer this question, we assessed the number of genes in each model not within two standard deviations of the mean in CF sputum, individually and in each possible combination (Fig. 5). In combination, the SCFM2 and acute mouse model outperforms all other pairs of models; only 358 genes are not mimicked by either the SCFM2 or the acute mouse model (compared to LB and the acute mouse model for instance which failed to capture 692 genes). There were 211 genes that were missed by every model we studied. These *“*elusive genes*”* include several genes whose expression is known to change via mutation that are common in P. aeruginosa lung-adapted strains, including genes involved in alginate production, pilus biosynthesis, and multidrug efflux.

Since it is unsurprising that some of these genes are poorly captured by the strain PAO1 which lacks these common CF lung mutations, we next tested whether a CF clinical strain, LESB58-SED21, which is well adapted to the CF lung, would perform better than PAO1 when grown in SCFM2. Interestingly, expression of 51 of the elusive 211 genes, including genes involved in the processes mentioned above (alginate production, pilus biosynthesis, and multidrug efflux), was within two standard deviations from sputum transcriptome means when LESB58-SED21 was grown in SCFM2 (Data Set S2). Further, the LESB58-SED21-in-SCFM2 model captured more genes than all models evaluated in Fig. 3 (AS_2_ = 89%, missing 579 genes shared with PAO1 [Data Set S2]). This result indicates that a CF clinical strain mimics certain features of P. aeruginosa CF lung infection better than PAO1, although it should be noted that PAO1 grown in model systems mimics the CF sputum gene expression of the majority of P. aeruginosa genes.

### Technical considerations of the framework.

A few details of our model evaluation framework are worth further exploration. First, we have normalized the read counts with the “variance stabilizing transformation” (VST) that is implemented in DESeq2, but other normalization methods are commonly used. We repeated the analysis with two other common normalizations, the “regularized log” (rlog) transformation also implemented in DESEq2, as well as the “trimmed mean of M values” (TMM) method as implemented in edgeR (33) (Fig. S5). The results were similar in all approaches; however, the standard deviations of sputum sample gene expression were greater using the TMM method, and though the qualitative results were similar (e.g., the SCFM2 and the CF airway epithelial cell model performed best), there was also a greater separation between the accuracy of sample types. We repeated the same process for metabolic subcategories of the MOPS-succinate model shown in Fig. 2B. Again, the qualitative results were similar for the three normalizations: “amino acid biosynthesis” performed poorly, and “biosynthesis of cofactors, prosthetic groups, and carriers” performed well (Fig. S5). As before, there was a greater spread among categories with the TMM normalization than with the two DESeq2 normalizations. Thus, while the normalization method influences model accuracy scores, the qualitative outcomes were very similar.

10.1128/mBio.03042-19.5 FIG S5 Model accuracy scores using two additional normalization methods. In the main text we used the variance stabilizing transformation (VST) provided from the DESeq2 package. Here, we show the effect of using the regularized log transformation from the DESeq2 package, as well as the popular TMM transformation from the EdgeR package on accuracy results. We first plot the accuracy scores for all models shown in Fig. 3 in the main text using regularized log transform (A) and using the TMM transformation (B). We then plotted the accuracy results for each functional category for MOPS-succinate as in Fig. 2B, but instead using the regularized log transformation (C), as well as the TMM transformation (D). Download FIG S5, PDF file, 2.97 MB. Copyright © 2020 Cornforth et al. 2020 Cornforth et al. This content is distributed under the terms of the Creative Commons Attribution 4.0 International license.

A second interesting consideration is whether genes that fell within a chosen standard deviation threshold actually had similar expression between the model and the sputum samples or whether they fell within this margin due to high variance among the sputum samples. To address this issue, we reanalyzed the data, restricting analysis to genes with low noise (with a low coefficient of variation, or the standard deviation divided by the mean, among the sputum samples). We found a small but statistically significant negative correlation between the coefficient of variation of expression for all genes among the clinical samples and number of standard deviations they are from the mean (*r* = −0.05, *P* = 2.2 × 10^−4^). Consistent with this finding, restricting the assessment to genes with low noise did decrease the genome-wide accuracy scores across the models, but it had a negligible effect on the relative accuracy scores of models compared to each other (Fig. S6). This result is likely because genes with noisy expression among the sputum samples increase the accuracy scores similarly for all models.

10.1128/mBio.03042-19.6 FIG S6 Model accuracy scores at various coefficient-of-variation cutoffs for sputum sample gene expression. Accuracy scores are plotted as in Fig. 3, but restricting only to genes with a coefficient of variation (standard deviation/mean) of less than 0.1 (A), 0.2 (B), or 0.3 (C). Restricting the assessment to genes with low noise does not qualitatively affect the overall ranking of the different model scores; however, the raw scores decrease. Download FIG S6, PDF file, 0.3 MB. Copyright © 2020 Cornforth et al. 2020 Cornforth et al. This content is distributed under the terms of the Creative Commons Attribution 4.0 International license.

## DISCUSSION

Despite microbiology’s heavy reliance on laboratory models, their accuracy has not been systematically evaluated. As a result, models are typically selected based on a researcher’s intuition, a laboratory’s expertise, or on limited experimental evidence, rather than on a solid biological rationale. Taking advantage of recent innovations in RNA-seq and sample preparation procedures that enable sequencing of human CF lung infection samples (24), we have begun to address this gap by focusing on a set of models used to study P. aeruginosa CF lung infections. By comparing 20 P. aeruginosa transcriptomes from human CF sputum, collected from clinics in the United States and Denmark and preserved immediately after expectoration, to several transcriptomes from commonly used laboratory models, we found that all models differed from the CF infections in important ways (Fig. 1). We propose a framework based on the deviation in expression among P. aeruginosa genes between model systems and human CF infection to provide an easily interpretable gauge of model performance (Fig. 2). Different models excelled at mimicking distinct biological functions in CF sputum (Fig. 3), and thus by combining the models we were able to accurately represent the expression of over 96% of P. aeruginosa PAO1 genes (Fig. 4). However, there were 211 genes that could not be captured by laboratory models using PAO1, but many of these genes could be captured when using a clinical strain.

Our initial survey of laboratory CF models already provides several useful insights. Surprisingly, gene expression in all models, even those such as LB that were not specifically designed to mimic P. aeruginosa CF infection, were similar overall to that in the CF lung. Indeed, over 80% of genes expressed in all models were within two standard deviations of the mean in CF sputum (Fig. 3B). These data support the notion that growing the lab strain PAO1 planktonically in LB is a viable model system for studying many aspects of CF lung infection and that many P. aeruginosa genes do not vary significantly in expression regardless of the genotype and growth environment. Also, somewhat surprisingly, the murine model performed no better than the *in vitro* models we tested. Murine infection models have become the gold standard for laboratory models because they are thought to approximate the chemical and physical environment of human infections, especially in terms of the host immune response (34). However, overall P. aeruginosa CF physiology is better captured by SCFM2 than by the murine lung infection model we tested (Fig. 3). Although it is clear that SCFM2 performs better overall than the mouse model by our accuracy score approach (Fig. 3B), there are functional categories for which the mouse model outperforms SCFM2; for instance, “porins” are more accurately represented in the mouse model than in SCFM2. Thus, while assessing overall accuracy scores is important, it is critical to also assess functional categories, pathways, and genes since it is likely that even the best overall models will not be superior in all cases. It is also important to point out that the mouse pneumonia model we evaluated here may be more accurate for non-CF pulmonary infections because it is an acute, rather than chronic, infection model. This acute murine model, when combined with the SCFM2 model, had expression within two standard deviations of the clinical sample means for all but 358 P. aeruginosa PAO1 genes (Fig. 5), better than any other model pairing.

Our work also identified 211 genes that were not expressed similarly to CF sputum when P. aeruginosa PAO1 was grown in any of the tested models. The expression of many of these “elusive genes” is known to change due to mutations accumulated during chronic lung infection, including genes involved in twitching motility, alginate production, and multidrug efflux. The fact that 51 of the 211 “elusive” genes (including most genes in these categories) were captured using the CF clinical isolate LESB58-SED21 grown in SCFM2 indicates that laboratory CF models may be improved for specific functional categories merely by using clinical strains rather than standard reference strains. It should be pointed out that LESB58-SED21 is a Liverpool epidemic strain from the United Kingdom (35) and was not an infecting strain in the CF sputum samples used in this study. Thus, it is not necessary to use a strain collected from the same clinic as the sputum samples in order to mimic CF lung-adapted gene expression profiles. Finally, while it is clear that using a clinical strain can be advantageous for studying specific functions such as mucoidy, this again does not seem critical for many aspects of P. aeruginosa physiology since the LESB58-SED21 strain in SCFM2 did only somewhat better than PAO1 in SCFM2 (missing 579 versus 681 genes shared by the two strains, *P* > 0.05 [*t* test]).

Our framework also provides strategies in addition to using CF-adapted strains for improving model systems. For example, SCFM2 performed poorly in the “polyamine biosynthesis” sub role, with some of the genes involved in biosynthesis of the polyamine spermidine expressed higher in SCFM2 than in CF sputum (*speD* and *speE*). Since SCFM2 does not contain spermidine, one can hypothesize that the addition of spermidine to SCFM2 would result in reduced expression of genes in the “polyamine biosynthesis” sub role, thus yielding a more accurate model. While this approach may currently be most useful for genes that respond to known regulatory cues, such as genes encoding well-understood biosynthetic and catabolic processes, we anticipate it will be useful for genes of unknown function as additional transcriptomic data become available to inform approaches such as gene interaction networks and functional annotations.

Of course, as we and others have proposed (36–38), interactions between microbes may also be an important modulator of P. aeruginosa gene expression in the CF lung. Although there is no definitive evidence of microbial interactions in the human CF lung, our approach will also be useful to determine whether the presence of commonly cooccurring microbes can improve the accuracy of P. aeruginosa model systems. In particular, we hypothesize that the addition of other microbes to our models will allow many of the elusive genes to be better mimicked, ultimately providing evidence for interactions in the CF lung. Finally, the addition of human cells such as neutrophils to the *in vitro* model systems may provide a step forward in defining the signals and cues that drive P. aeruginosa gene expression in the CF lung.

As more transcriptomes become available for each model, we will be able to assess not only the mean accuracy score of any set of genes but also the distribution of these scores, as sometimes the mean will miss interesting features of transcriptome variation. For simplicity, we have used the z-score of normalized counts, which is based on the normal distribution, but other distributions are also viable. For instance, the t-distribution is typically used in place of a normal distribution when the number of samples is small; however, we used z-scores because the number of available CF sputum sample transcriptomes is quickly growing, and we also wanted to avoid the accuracy score explicitly depending on the number of available transcriptomes. Another distribution commonly used in gene expression analysis is the negative binomial (39), but we felt the normal distribution was more appropriate as an initial framework. Also, we have focused on genes whose expression in the models fall within two standard deviations (AS_2_) of the mean in the clinical population primarily out of convention, since two standard deviations for each gene encompasses expression in ∼95% of the CF sputum samples. However, there may be instances in which it is valuable to be more stringent. For example, if a gene or function is mimicked by a large number of model systems, one could further explore the models using more stringent criteria such as AS_1.5_ (although this would have a false-negative rate of approximately 13%). Ultimately, the ease with which our framework can be adapted provides researchers with the ability to rapidly and quantitatively compare multiple models for any trait of interest. Lastly, we expect that other approaches, including proteomics, metabolomics, or microscopy, will eventually be integrated into a comprehensive model evaluation approach.

### Conclusions, caveats, and future directions.

We have proposed a simple computational framework that can be used to aid experimentalists in selecting laboratory infection models. For example, if one were studying bacterial metabolism in relation to CF infection, SCFM2 is a better model than the airway epithelial cell model (Fig. 4A and B). However, if one were specifically studying fatty acid and phospholipid metabolism, then the epithelial cell model may be a better choice. Similarly, our results suggest that using clinical isolates may be the only way to accurately reproduce the gene expression profiles for some genes.

We focused on CF lung infection models because of the availability of clinical samples and developed models; however, we did not conduct an exhaustive characterization of CF infection models. Such an evaluation would require systematically sweeping a range of important experimental variables, including the strain or host genotype, coinoculated microbial community, the physiology of the bacteria before inoculation, and the time point after inoculation that the sample is taken. Any of these factors may impact bacterial physiology and behavior, and we consider each separate perturbation to be a different experimental model. For instance, here, we focused on *in vitro* samples collected during mid-logarithmic growth, but LB medium samples taken at 7 h versus 10 h may have distinct gene expression signatures, and so we consider these different models. It is also important to note that different laboratories may conduct *in vitro* experiments slightly differently even when using the “same” experimental system and protocol. Further, differences in library prep processes can potentially impact accuracy score calculations and comparisons between models. Some library prep kits are stranded, and others are nonstranded; in the present study, we compared models without using strand information because not all samples were prepped with stranded RNA-seq library prep kits. Lastly, downstream analysis, including the software and normalization method used, may also impact the accuracy score of a system. All of these issues must be controlled for before definitive conclusions can be reached about the superiority of one model over another for a particular biological question.

Since Robert Koch’s first use of guinea pigs as a model for TB infection, microbiology has relied on a range of laboratory models (40). However, there is no system to comprehensively evaluate a model’s accuracy. The framework we propose here is a step toward a general model evaluation framework that is applicable to any microbial model and will only become more powerful as functions for unknown genes are discovered. Our approach can also easily be extended in the future to community-wide functionality in polymicrobial communities, rather than simply the functions of its individual members.

## MATERIALS AND METHODS

### Data.

SRA accessions for the raw reads from all analyzed sequencing files are provided in Data Set S1 in the supplemental material. Data that have not been published have been uploaded under accession number PRJNA576508. Expectorated CF sputum samples for this study were collected in RNAlater from Emory–Children**’**s Center for Cystic Fibrosis and Airways Disease Research by the Cystic Fibrosis Biospecimen Laboratory as previously described by our group (24) with IRB approval (Georgia Tech approval H18220).

### RNA extraction and preparation of sequencing libraries for RNA-seq.

*In vitro* and human samples were prepared as previously described (24) with a few modifications for the human samples. For the human sputum samples, expectorated sputum was collected from adult patients that were clinically stable and immediately added to RNAlater and stored at 4°C overnight and then at –80°C. Samples in RNAlater were thawed on ice and centrifuged at 4°C for 30 min at 10,000 × *g*. RNAlater was removed from the sample, and the sputum was transferred to bead-beating tubes containing a mixture of large and small beads (2-mm zirconia and 0.1-mm zirconia/silica, respectively). *In vitro* cultures stored in RNAlater were pelleted, resuspended in 1 ml of RNA-Bee (AMS Biotechnology), and transferred to bead-beating tubes. Samples were resuspended in RNase and DNase-free TE buffer (Acros Organics) and lysozyme (1 mg/ml, final concentration) and lysostaphin (0.17 mg/ml, final concentration) were added to each sample. Samples were incubated at 37°C for 30 min to enzymatically lyse cells. RNA-Bee was added to each sample, and samples were lysed mechanically by bead beating three times for 30 s, placing the tubes on ice for ≥1 min between each homogenization. Portions (200 μl) of chloroform per 1 ml of RNA-bee were added, and the tubes were shaken vigorously for 30 s and then incubated on ice for 5 min or overnight to allow phases to partition. The samples were centrifuged at 12,000 × *g* for 15 min at 4°C to separate the aqueous and organic phases. The aqueous phase from each tube was transferred to a new microcentrifuge tube to which 0.5 ml isopropanol per 1 ml of RNA-Bee was added in addition to 20 μg of linear acrylamide, and the tubes were incubated at –80°C overnight. Samples were thawed on ice and centrifuged at 12,000 × *g* for 30 min at 4°C. Pellets were washed with 1 ml of 75% ethanol, air dried for 5 min, and resuspended in 100 μl of RNase-free water. The RNA concentration for each sample was determined with a NanoDrop spectrophotometer (Thermo Fisher Scientific). rRNA was depleted using a RiboZero Gold bacteria kit (Illumina) for the *in vitro* samples and a RiboZero Gold epidemiology kit (Illumina) for the human samples and purified by ethanol precipitation using linear acrylamide to help precipitate the RNA. The depleted RNA was fragmented for 2 min with the NEBNext Magnesium RNA fragmentation module kit and cDNA libraries were prepared using the NEBNext multiplex small RNA library prep kit (New England Biolabs) according to the manufacturer’s instructions. Libraries were sequenced at the Molecular Evolution Core at the Georgia Institute of Technology on an Illumina NextSeq500 using 75-bp single-end runs.

### Bioinformatic analyses.

RNA-seq reads were trimmed using Cutadapt 1.13 (51), using a minimum read length threshold of 25 bases. The non-P. aeruginosa “decoy” species were identified from previous work using CLARK 1.2.3 with an abundance cutoff 2% in at least one human sample (see Data Set S1 in the supplemental material) (24, 41). We built a metagenome using these species by downloading at least one genome from each of the 53 species identified, in addition to S. epidermidis, from the National Center for Biotechnology Information as previously described (24). We expect that these non-P. aeruginosa reads map to the similar decoy species better than to P. aeruginosa. For all samples, reads were mapped to this metagenome using Bowtie 2.2.6 with the default parameters for end-to-end alignment (42). We removed the reads that mapped to non-P. aeruginosa species from our trimmed reads files using Seqtk (43) and mapped the remaining reads to PAO1 (NC_002516.2, NCBI Assembly: GCF_000006765.1, gff-spec-v1.21). Reads were counted for using Rsubread 1.26.1 using default options and an SAF input file composed of all genes without decimal points in their locus tags (44). Because not all models had data with strand information available, comparisons were conducted without strand specificity; however, this did not qualitatively impact results compared to strand-specific analysis for differential expression and enrichment analyses. For the PCA in Fig. 1, we used a subset of 2,606 genes such that each had least one read mapping to it for all the samples (Data Set S2). For PCA and calculation of model accuracy, DESeq2’s variance stabilizing transformation function was used (v1.20.0) (39). Differential expression between the Denmark and U.S. clinics (Data Set S2) was also determined using DESeq2. All figures were created using ggplot2 (45). The R package fgsea was used for enrichment analysis using the stats score –log(*P* value) × sign(log_2_FC) and nperm = 1,000 (46). The sunburst plots in Fig. 2 and 4 were generated using R package ggsunburst (47). The UpSet plot in Fig. 5 was prepared by altering the UpSetR R package (48).

### Mammalian cell culture.

Immortalized homozygous CFTR ΔF508 CFBE41o^–^ human bronchial epithelial cells (obtained from J. P. Clancy, Cincinnati Children’s Hospital) were maintained in a humidified incubator at 37°C and 5% CO_2_ in minimal essential Eagle medium (MEM) containing phenol red (Gibco) supplemented with 10% fetal bovine serum (Gemini Bio-Products), 0.5 μg/ml Plasmocin prophylactic (InvivoGen), 2 mM l-glutamine, 5 U/ml penicillin, and 5 μg/ml streptomycin (Sigma) (19). CFBE41o^–^ human bronchial epithelial cells are not on the commonly misidentified list, and cells were tested quarterly for mycoplasma using a Southern Biotech mycoplasma detection kit. CFBE41o^–^ epithelial cells were seeded at near confluence on transwell permeable-membrane supports (Costar). After attachment and confluence, CFBE41o^–^ epithelial cells were differentiated at air-liquid interface for 1 week (49).

### Infection of differentiated respiratory epithelium.

For bacterial biofilm growth on biotic surfaces, CFBE41o^–^ human bronchial epithelial cells were inoculated in duplicate with P. aeruginosa that was prewashed in MEM lacking phenol red supplemented with 2 mM l-glutamine at a multiplicity of infection of approximately 25. A strain of P. aeruginosa PAO1 carrying a multiple copy plasmid that constitutively expresses *gfp* (pSMC21) was used (19). After 1 h of attachment, nonattached bacteria were removed, and the apical medium was adjusted to 0.4% l-arginine. After an additional 5 h, biofilms were processed for RNA. P. aeruginosa strain PAO1 RNA was isolated from biofilms grown for 6 h on differentiated CFBE41o^–^ cells by phenol-chloroform extraction using RNA-Bee and 0.1-mm zirconia/silica beads in a BeadBeater (BioSpec Products) (50). RNA was precipitated with isopropanol and linear acrylamide, and RNA pellets were washed by ethanol precipitation (50). RNA was treated with Turbo DNase (Ambion) and purified by an RNA Clean and Concentrator (Zymo Research). RNA concentration was measured by using a NanoDrop apparatus. DNA removal was confirmed by 260/280 and 260/230 ratios and by PCR for the *rplU* gene. RNA integrity was determined by agarose gel electrophoresis and visualization of 5S, 16S, 18S, 23S, and 28S bands. RNA-seq library preparation and sequencing were performed by the Health Sciences Sequencing Core at Children’s Hospital of Pittsburgh. RNA concentration and integrity was confirmed by fluorometric quantification (Qubit) and Tapestation analysis (Agilent). RNA was rRNA depleted using Ribo-Zero Epidemiology, and sequencing libraries were prepared using a Truseq stranded total RNA kit (Illumina). Single-end sequencing was performed on a NextSeq 500. For CFBE41o^–^ human bronchial epithelial cell-P. aeruginosa biofilm coculture samples, approximately 75 million 75-bp reads were obtained.
