# Supplementary figures and images for: Quantitative Framework for Model Evaluation in Microbiology Research Using Pseudomonas aeruginosa and Cystic Fibrosis Infection as a Test Case
Source: mBio. 2020 Jan 14;11(1):e03042-19. doi: 10.1128/mBio.03042-19 (PMC6960289; doi:10.1128/mBio.03042-19)

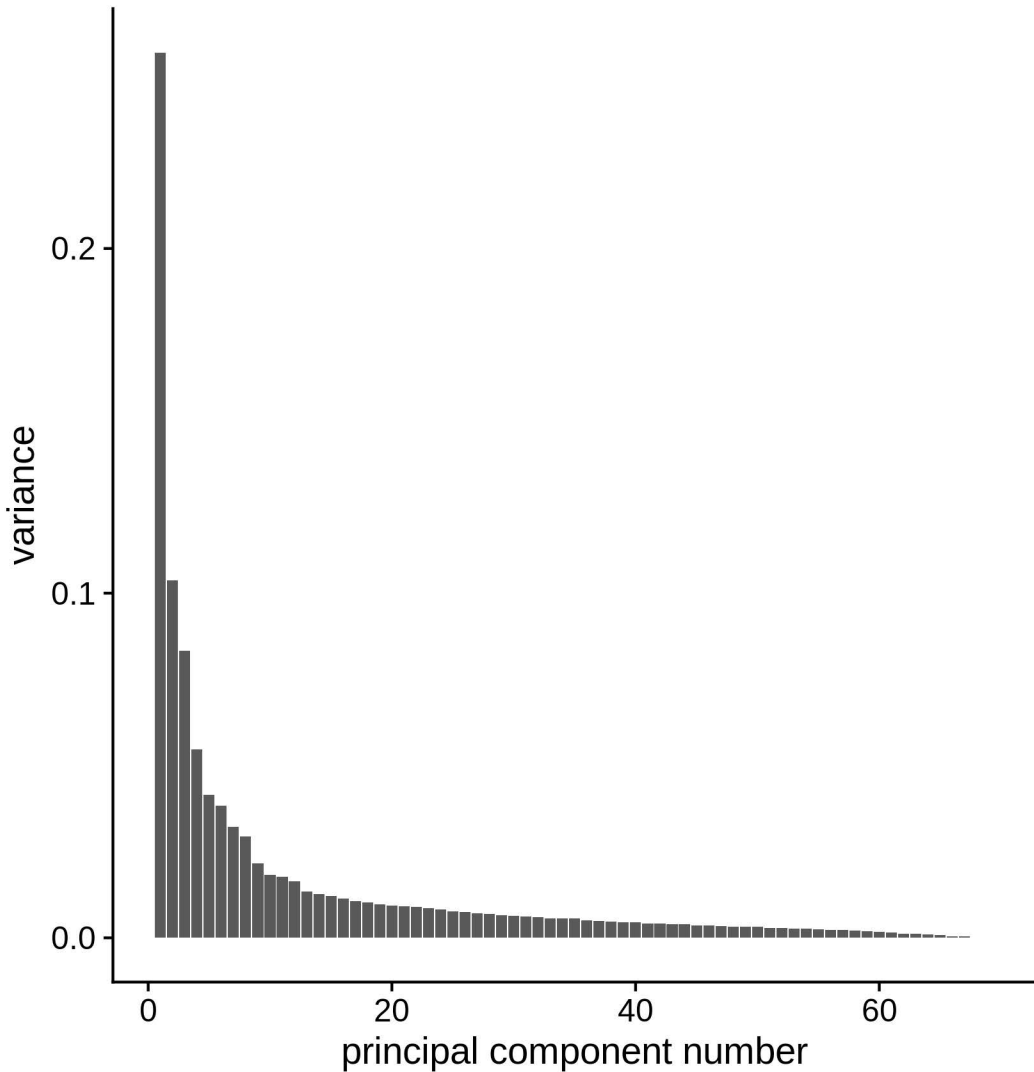

Supplement: FIG S1 [file mBio.03042-19-sf001.pdf]

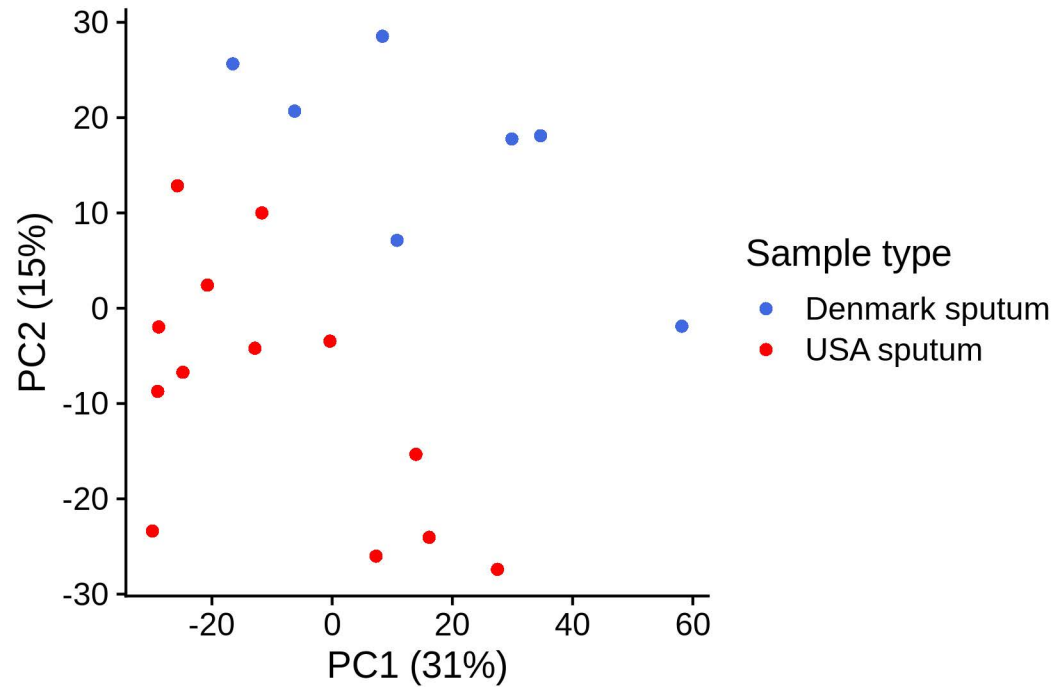

Supplement: FIG S2 [file mBio.03042-19-sf002.pdf]

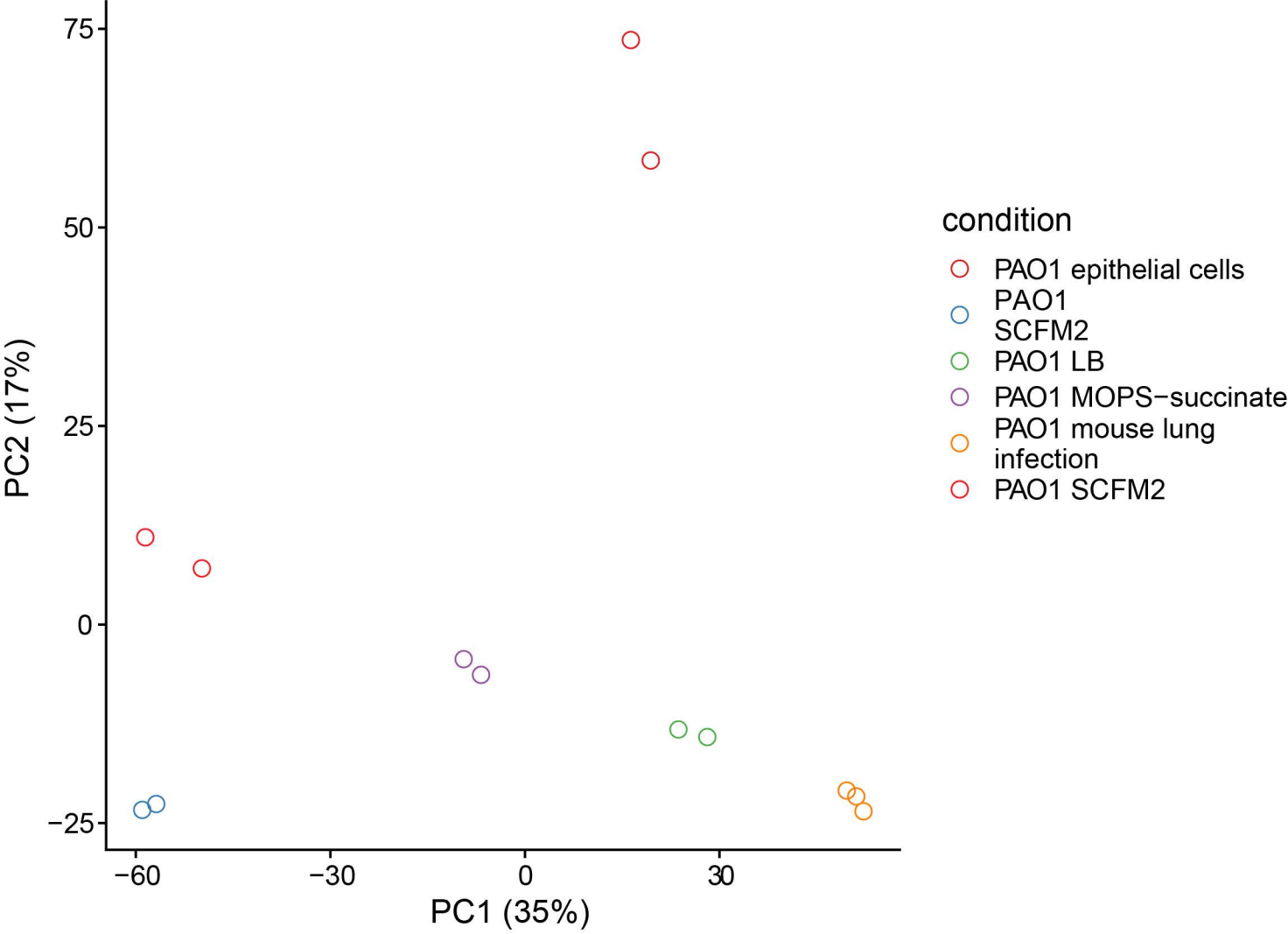

Supplement: FIG S3 [file mBio.03042-19-sf003.pdf]

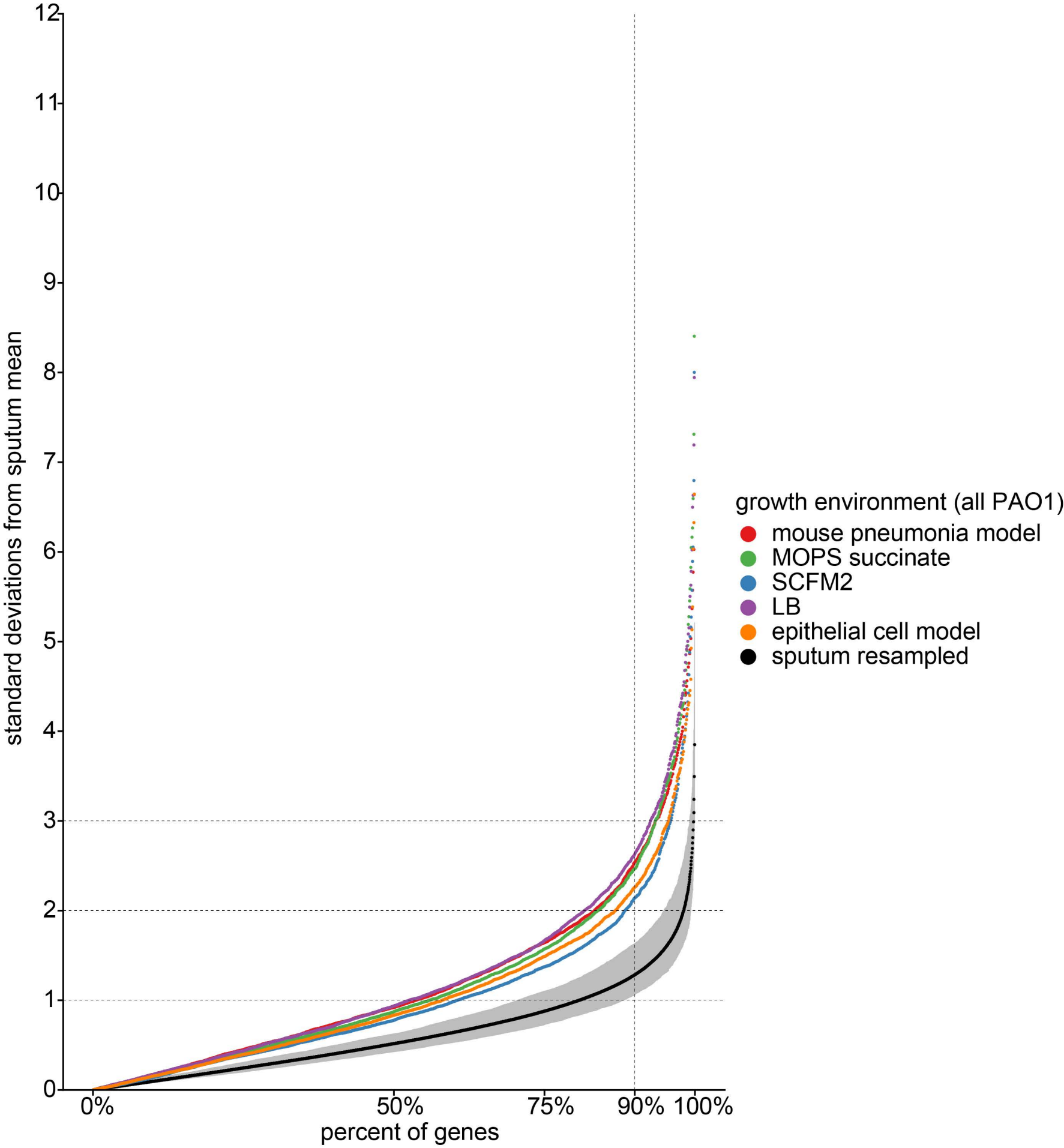

Supplement: FIG S4 [file mBio.03042-19-sf004.pdf]

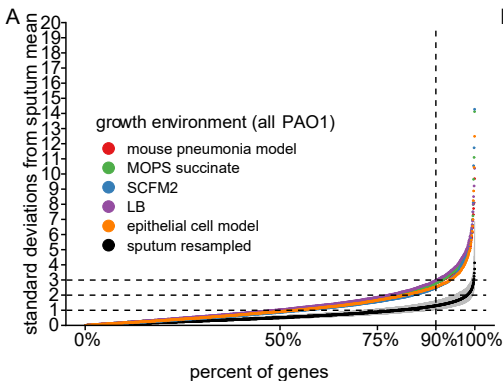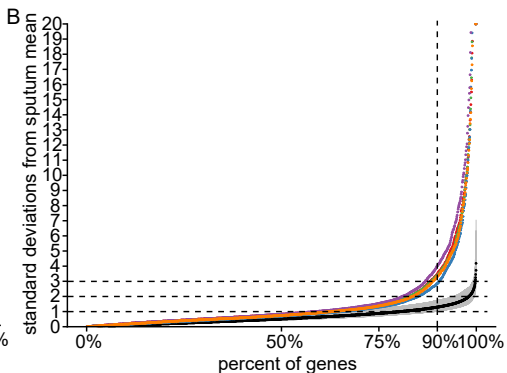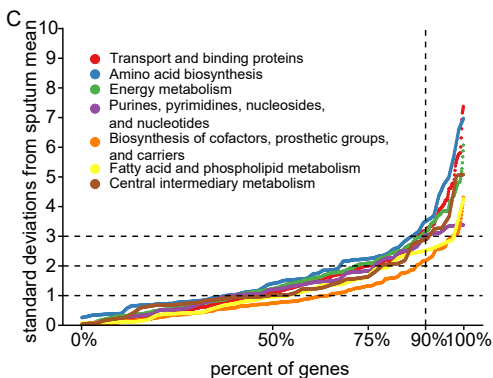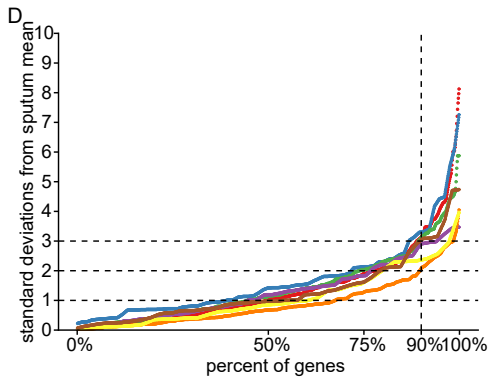

Supplement: FIG S5 [file mBio.03042-19-sf005.pdf]
