# Supplementary material for: Quantitative Framework for Model Evaluation in Microbiology Research Using Pseudomonas aeruginosa and Cystic Fibrosis Infection as a Test Case
Source: mBio. 2020 Jan 14;11(1):e03042-19. doi: 10.1128/mBio.03042-19 (PMC6960289; doi:10.1128/mBio.03042-19)
Supplement: FIG S6 [file mBio.03042-19-sf006.pdf]

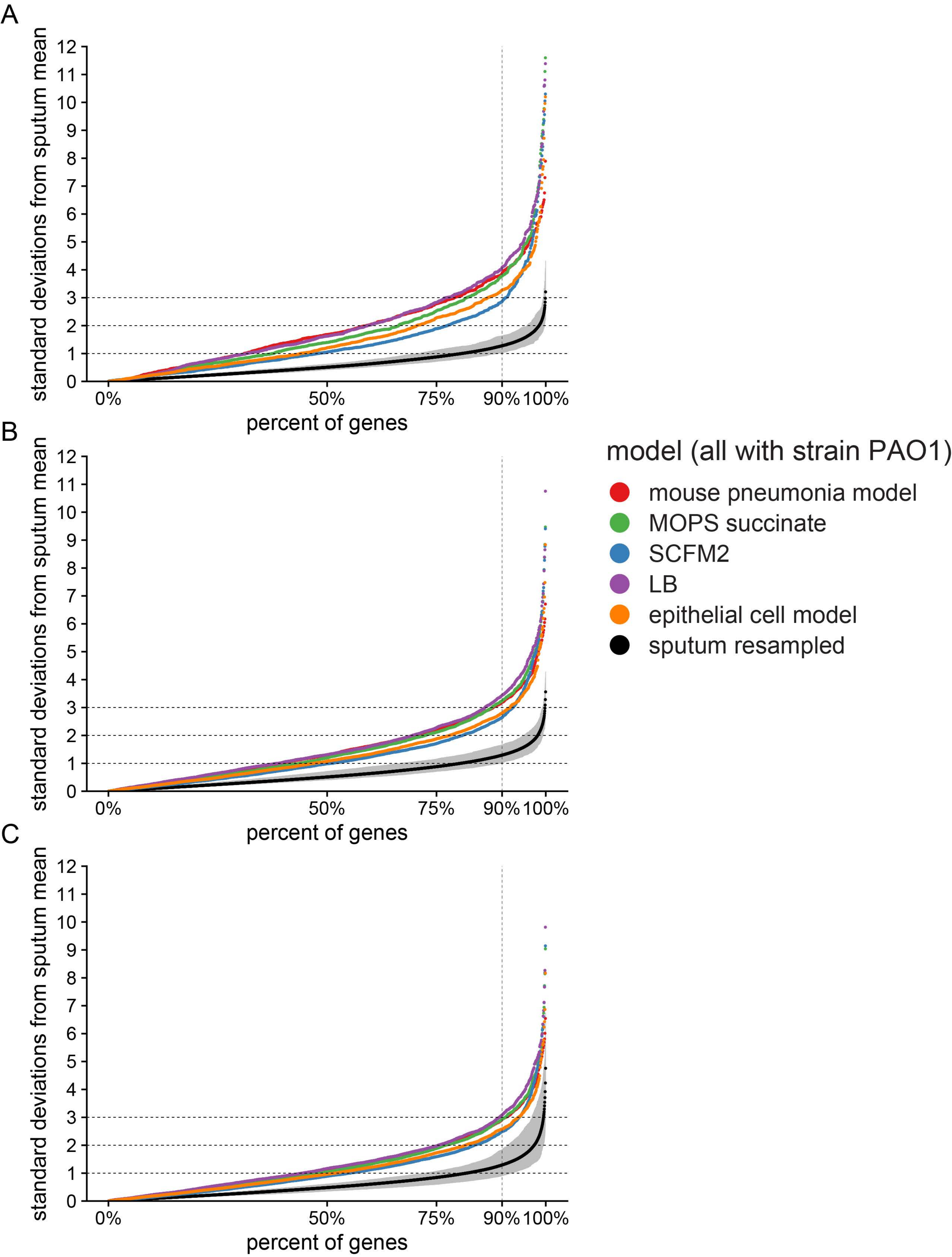
A
B
C
standard deviations from sputum mean
0
1
2
3
4
5
6
7
8
9
10
11
12
0%
50%
75%
90%
100%
percent of genes
model (all with strain PAO1)
mouse pneumonia model
MOPS succinate
SCFM2
LB
epithelial cell model
sputum resampled
